# Traumatic spinal cord injury in Saudi Arabia: a review of the literature

**DOI:** 10.11604/pamj.2013.16.104.2902

**Published:** 2013-11-18

**Authors:** Asirvatham Alwin Robert, Marwan M Zamzami

**Affiliations:** 1 Research Center, Medical Affairs, Sultan Bin Abdulaziz Humanitarian City, Riyadh, Saudi Arabia; 2 Department of Orthopedic Surgery, College of Medicine, King Saud University, Riyadh, Saudi Arabia

**Keywords:** Traumatic spinal cord injury, trauma, Saudi Arabia

## Abstract

Traumatic Spinal Cord Injury (TSCI) is a condition where the neural elements suffer acute trauma, resulting in short-term or permanent sensory and motor problems. An understanding the underlying structural and functional biological repairs of the TSCI mechanisms has intensely increased over the last two decades. However, compared with the other fields in medicine, the present degree of treatment and care for TSCI are quite unsatisfactory. The Kingdom of Saudi Arabia (KSA), the largest country in the Middle East, occupies nearly four-fifths of the Arabian Peninsula with a population of over 28 million. It also has the distinction of having one of the highest rates of spinal cord injuries in the world. However, research on TSCI has been very limited. Therefore, studies on the long-term incidence of TSCI in Saudi Arabia are vital and most essential to identify the high-risk groups, create awareness, establish trends, predict the needs, and thus contribute to effective health care planning of this condition. In this review, we discuss various aspects of TSCI in Saudi Arabia from the available literature.

## Introduction

Traumatic Spinal Cord Injury (TSCI) is a devastating neurological injury, causing paralysis, sensory loss and sphincter disorder in different degrees and indirectly imposes a significant burden on the health care system [1, 2]. Based upon the location and degree of injury, and irrespective of the advanced medical management, the probability of death during the pre-hospital as well as the acute phase is still present [3].

In the developed countries, TSCI has been studied in great detail, and several research papers have been published over the last few decades. However, most of the research published considers only a limited section of the world's population. Although research reveals that more than 80% of the world′s population is spread across more than 100 developing countries [4], information regarding TSCI from these countries is still meager [4]. Besides, an established national spinal trauma or Spinal Cord Injury (SCI) registry is lacking among the developing countries. Also, detailed records of population-based data on TSCI are also limited from most of the developing countries. Further complications arise as the majority of the hospitals do not maintain meticulous medical records [5]. Further, most of the data published are surveys from single center hospitals representing fewer than 15 developing countries [6–9].

The Kingdom of Saudi Arabia (KSA), the largest country in the Middle East, occupies almost four-fifths of the Arabian Peninsula with a population of over 28 million [10]. The United Nations has predicted that the population of Saudi Arabia will raise up 39.8 million by 2025, and 54.7 million by 2050 [11]. The government of Saudi Arabia has assigned great importance to health care services, as a result, health and health services have improved significantly in terms of quantity and quality, over the past few decades. However, health-related researches such as SCI researches continue to face many challenges. Nevertheless, SCI remains one of the most indispensible social and economic medical issues in the Kingdom. Living with disability becomes a harsh reality for many SCI patients, who encounter different problems at different stages in life [12]. Compared with the developed countries, very limited research on TSCI has been conducted in Saudi Arabia. The aim of this review is to explore the many and varied aspects of SCI in Saudi Arabia from the published literature.

## Methods

With the help of a senior researcher, a literature search was conducted, to investigate in the National Library of Medicine (PubMed) and Ovid Medline databases. General search engines were also employed to access non-peer reviewed professional and specialist guidelines and workshops on SCI websites, limiting the search to English and Arabic publications only. Selection of papers was done by reviewing their titles and abstracts using the additional references identified from their reference lists.

### Current status of knowledge

#### Prevalence and Incidence

The Ministry of Health (MOH), Saudi Arabia, has recorded one of the highest rates of spinal cord injuries in the world, mostly resulting from Road Traffic Accidents (RTAs). However, the information available is too limited to estimate the accurate incidence of TSCI in Saudi Arabia. According to a recent hospital-based, retrospective study, the incidence rate of TSCI was reported to be 2.1 per million. This study was conducted on 307 TSCI patients at the Riyadh Military Hospital (the main medical services provider for military personnel) between 2003 and 2008 [13]. Another hospital-based study was done at Riyadh Central Hospital, between 1979 and 1984 involving the complete records of 377 patients who sustained traumatic injury [14]. Apart from the smaller sample-sized single hospital-based, retrospective studies, there is no published data available on the prevalence and incidence of SCI [13, 14]. However, over the last decade, in a project submitted by AboAbad to the Department of Orthopaedic Mechanics University of Salford, Salford, UK, 1999, the annual incidence of SCI was revealed to be 62.37 per million [15]. Further, an increase in the incidence of SCI (including traumatic and non-traumatic SCI) from 1990 onwards was reported, which reached its peak in 1994. The study also reported that between 1990 and 1994 (population 78,000), the prevalence of SCI in Saudi Arabia was 627 patients per million [15]. Recently, the project submitted by Al-Shammari to the University of Birmingham, Birmingham, UK showed the incidence of SCI in Saudi Arabia as 38 per million (including traumatic and non-traumatic SCI, during 2000-2010, population 37,000) [16]. The difference in the rate of incidence of SCI in Saudi Arabia may be caused by variations in the definition, inclusion criteria, classification and procedures employed in patient identification, geographical and cultural differences, as well as differences in the pre-hospital and hospital treatment available [13, 17–19]. However, these projects reports reveal evidence that proves Saudi Arabia has the highest incidence rate of SCI (62.37 and 38 per million) compared with most other countries, over the past years. The national SCI data of the other countries reveal North America at 40 per million, Western Europe at 16 per million and Australia at 15 per million ranking below. Further, extrapolated regional data revealed the incidence in other regions as follows: Asia-Central (25 per million), Asia-South (21 per million), Caribbean (19 per million), Latin America, Andean (19 per million), Latin America, Central (24 per million), Latin America-Southern (25 per million), Sub-Saharan Africa-Central (29 per million) and Sub-Saharan Africa-East (21 per million) respectively [20]. The data published on the prevalence of SCI was limited when considering the range of 236-1800 per million inhabitants as representing a worldwide estimate [17]. However, an estimated incidence rate from the Middle East, Jordanian, Qatari and Turkish incidence data is about 15 TSCIs per million per year; however, it is most likely underestimated [13].

It is noteworthy that the incidence quoted for Saudi Arabia is at the higher end which makes long-term incidence studies in Saudi Arabia very important and essential to identify the high-risk groups, create awareness, establish trends and predict the requirements, and thus contribute to effective health care planning in TSCI.

### Etiology

In Saudi Arabia, RTAs spinal cord injuries are still the primary cause of SCI, with a high percentage of total injury at the onset of rehabilitation, particularly in the young adult section of car drivers [21]. A study between 1971 and 1997 reported that 564,762 people had died or suffered injuries in road traffic accidents [22]. Further, a recent report stated that during 2010 to 2011, traffic accidents claimed 7,159 lives, while more than 40,000 were injured in more than half a million (54,400) traffic accidents that took place in Saudi Arabia [23]. It is clear that the occurrence of RTAs is very high in Saudi Arabia and they are the major causes of SCI, a finding confirmed by a hospital-based study, which showed that 79.2% of patients admitted for spinal injuries had sustained their injuries in RTAs [22]. Other studies also indicated that more than 80% of the SCI patients had sustained injuries due to RTAs followed by falling from heights [24, 25]. In the literature it has been reported that RTAs are in fact, the second major health problem after infectious diseases [13, 21, 26]. The frequency of the RTAs caused by four-wheeled vehicles is one of the highest globally reported RTA statistic [13]; also, particularly during Ramadan, the incidence of RTAs rises higher than in other months [27, 28]. This may be a result of a few reported reasons such as dietary habit changes and minimal sleep duration during the period of the Ramadan fasting [29, 30].

Studies reported that the causes of SCI vary from one country to another, for instance the primary cause of SCI in South Africa which is violence, in Western Europe is the four-wheel motor vehicles, and Southeast Asia is the two-wheel and “nonstandard” vehicles, whereas falls from trees and rooftops are the major causes of SCI in Southern Asia and Oceania. However, following RTAs, falls remain the second most common cause worldwide, although in some regions they are even more common than the RTAs, such as in Nepal, where 75% of the SCIs are due to falls from heights [31, 32].

### Gender differences

Research has shown that due to variations in the socio-economic levels and cultural backgrounds, the male/female ratio of SCI varies significantly between different countries. In Saudi Arabia, in particular, women are not permitted to drive cars and cannot participate in outdoor activities because of the socio-economic levels and cultural background [33]. The World Economic Forum 2009 global gender gap had reported and ranked Saudi Arabia 130th out of 134 countries for gender parity [33]. Therefore, women are less involved in RTAs. Many studies from Saudi Arabia also reported that men are at more risk for SCI than women. In fact more than 80% of the SCI patients in Saudi Arabia are men, particularly those in the younger age group [13, 21, 24, 34, 35]. Studies also reported that TSCI primarily affects males between 18 and 32 years of age, RTAs being the primary cause [36]. Further, Dryden et al., reported that the risk of traumatic spinal cord injury is 2.5 times higher in the rural than in the urban areas [37].

### Age at injury

A recent study from Saudi Arabia reported that the TSCI frequency was higher in the 21-30 (40%) and 31-40 (19.7%) age groups and lower in the 71-80 (2.2%) age group [21]. Also, Al Jadid, (2013) reported that a higher frequency of TSCI occurred in the 16-30 age group [24]. Another study stated that the majority of the SCI patients belonged to the age groups of 21-30 years (40.4%) and 31-40 years (33.3%) [25]. The higher risk group was 20-30 years in most of the studies [21, 25, 34].

Studies reported that SCI before the age of 15 years is a relatively infrequent occurrence, although it can have major psychological and physiological consequences [38]. The mechanism of injury is different based upon the age at the time of injury; neurological recovery appears to be better in the younger population compared with adults [38].

### Level of injury

The anatomical level of the SCI is distinguishable into high (cervical) and low injuries (thoracic, lumbar and sacral) [17, 39]. The neurological level of injury is defined as the most caudal segment of the spinal cord that has normal function [17, 39]. However, it is difficult to locate the neurological level of SCI without clinical examination of the patients.

A few studies have reported the neurological level of SCI in the Saudi population. A study on Saudi male SCI patients reported 43.9% with cervical injury followed by 40.4% with thoracic injury and 3.5% with lumbar injury [25]. The majority of cervical injuries occurred in the age group of 21-40 years. Also, a recent study reported that the cervical cord was the most common site of injury, accounting for 34% of cases of TSCI among the males. Further, a study on females also reported that the most frequent (82%) level of injury was at the thoracic vertebrae followed by the cervical (12%) and lumbar (6%) [34].

### Quality of life

The World Health Organization defines “Quality of Life” (QoL) as the “individual's perception of their position in life in the context of the culture and value systems in which they live, and in relation to their goals, expectations, standards and concerns” [40]. The SCI is a very real problem in the third world countries and could be a crippling medical problem, especially for the patients in performing their daily normal and physiological life rhythms [25]. However, with increasing alertness and developments in the management of complications from SCI, individuals are able to live longer and more satisfying lives [41].

Compared with the developed countries, the QoL among the Saudi SCI patients is not satisfactory. A study on the male SCI patients reported that urinary incontinence was managed by intermittent catheter (28%), indwelling catheter (17.5%), suprapubic cystostomy (15.8%), condom (12.3%) and continent (14.1%) [25]. Pressure sores were common and complications resulted in urinary tract infections in 80.7% of the patients. In the case of managing bowel incontinence, 75.4% used suppositories, 12.3% could perform manual evacuation, 8.8% were assisted by enema and only 3.5% were continent. In terms of the genitourinary complications, more than three-quarters of the patients (80.7%) had developed recurrent urinary tract infections, 50.9% developed pressure sores during the course of their life and 21.1% began to contract chronic diseases such as diabetes and hypertension [25]. Spinal cord injury was a major cause for these and significantly influenced patients′ employment and career. Other important factors affecting the patient′s QoL were financial status, employment, equipment supply and social isolation [25]. Another study on the female SCI patients reported that more than 76% of them developed pressure sores, and almost 34 (62%) had already begun to contract chronic diseases, such as diabetes, hypertension and others [34].

### Psychological factors

Depression and anxiety disorders and/or symptoms are commonly reported post SCI. Although a conceptual distinction exists between depression and anxiety, clinically differentiating between the two constructs has proven difficult, as people who experience anxiety are often depressed as well [42]. People who have experienced SCI, on average, are subject to higher levels of distress and lower levels of life satisfaction compared with the general population [43]. In Saudi Arabia, limited studies have been conducted on SCI and psychological well-being. A recent study reported that compared with the male TSCI patients, females revealed significantly elevated levels of anxiety and depression scores [35]. Further, this study also reported that the correlation between the education level, anxiety and depression showed that patients with a university education experienced higher degrees of anxiety and depression than patients with a lower education level [35]. The TSCI patients with pain reported more anxiety and depression than patients who did not experience pain [35].

### Hospital length of stay

In Saudi Arabia, a recent report indicated that patients with TSCI had a hospital Length of Stay (LoS) of 46.2 days, and the males compared with the females among the traumatic spinal cord injured patients endured a longer duration of hospitalization [21]. This hospital-based study also reported that the LoS of the Saudi patients was higher than that of the non-Saudis (other Arab population) [21]. However, the LoS differed in reports from various other countries. The mean values were found to be 20-74 days in the USA [44], 56-61 in Australia [45], 91-143 in Italy [46], 154-240 in the Netherlands [47, 48], 198-222 in Spain [49] and 267 in Japan [50]. The LoS of the Saudi TSCI patients is comparable with that of the reports from the USA and Australia [21].

In cases of SCI, acute care is generally completed within a few months of the injury and the neurological recovery reaches a plateau, marking the time for the patient to be discharged [51]. However, in domestic cases, many patients seek several hospitals for admission, instead of returning to normal life. A long hospital stay utilizes medical resources and leads to substantial social loss [51].

It should be noted that the LoS in the hospital is a major contributor to the direct cost of SCI care. In their efforts to contain health care costs, providers have attempted to decrease the patients’ average LoS in the hospital. The assumption that has been reducing LoS enables cost savings [21, 52]. A study reported that reducing the LoS by even as less as one full day reduces the total cost of care on average by <3% [52].

### Medical care and rehabilitation

Medical and technological advances have comprehensive survival rates through the ever increasing effective acute clinical management for spinal cord injured patients, although the links between rehabilitation and recovery are less well understood [53]. Spinal injuries that are ignored or secondary to ‘overlooked’ diagnosis may result in severe medical and medico-legal problems. Although these are not unusual they are less frequently reported in the medical literature. In the developing countries, untreated or inadequately treated spinal injuries with late presentation are more frequently seen. Unfortunately, reports on such cases too are seldom published [54]. Rehabilitation Medicine has not yet become well developed in most of the developing countries and is often “confused with physiotherapy, rather than with the concept of a multidisciplinary approach”. The number of specialists trained in Rehabilitation Medicine or in SCI management is still very small in many developing countries [55].

Over the last three decades, the Ministry of Health (MOH), Saudi Arabia opened various rehabilitation centers for persons with disabilities and other nationalities in the country. However, the majority of these programs cover only physical, occupational, speech and hearing therapies, as well as prosthetic and orthotic services within the existing modern and sophisticated health care service system and infrastructure [56, 57]. Rehabilitation programs and facilities, as an intrinsic part of modern health care delivery services, have received due attention by the government authorities, with services being made available to all the citizens and residents. In the early 21st century, many such modern medical rehabilitation centers were started in a few MOH hospitals with good facilities. In addition, some private non-profit centers have also been opened, including the Sultan Bin Abdulaziz Humanitarian City [58]. At present, there are several rehabilitation hospitals/centers available in the Kingdom, mainly in the larger cities, such as the Rehabilitation Unit of Prince Sultan Military Medical City of Riyadh, Rehabilitation Unit of King Abdulaziz Medical City, National Guard (Riyadh), Rehabilitation Hospital of King Fahad Medical City (Riyadh), King Saud Medical Complex, Rehabilitation Hospital of Al-Hada Military Hospital (Taif) and Riyadh Care Hospital [10].

## Conclusion

In Saudi Arabia, there continues to be a dearth of meticulously conducted research on traumatic spinal cord injury. However, such research is highly essential to plan for appropriate management programs, effective implementation of primary prevention strategies and proper allocation of health resources for this traumatic condition.
